# Factors influencing the uptake of Voluntary HIV Counseling and Testing among secondary school students in Arusha City, Tanzania: a cross sectional study

**DOI:** 10.1186/s12889-015-1771-9

**Published:** 2015-05-02

**Authors:** Zawadi Sanga, Gibson Kapanda, Sia Msuya, Rose Mwangi

**Affiliations:** Department of Community Health, Kilimanjaro Christian Medical University College, Moshi, Tanzania; Kilimanjaro Christian Medical University College, KCMC-MEPI Project, Moshi, Tanzania

**Keywords:** VCT uptake, HIV test, Knowledge, Attitudes, Secondary school students

## Abstract

**Background:**

Voluntary HIV counseling and testing (VCT) is a key strategy towards HIV prevention yet, the uptake of VCT services among young people remains low. This study determined the factors that influence the uptake of VCT among secondary school students in Arusha City, Tanzania.

**Methods:**

A cross sectional study using quantitative methods was conducted. A multi-stage sampling method was applied to randomly select the secondary schools. Stratification, random and systematic sampling techniques were used to identify the study participants. Interviews were conducted using structured questionnaires. Data analysis was done using statistical package for social sciences version 16. Analytical statistics were done using odds ratio to measure strength of association between VCT uptake and independent variables. Association with p-value < 0.05 was considered significant. Binary logistic regression was used to identify predictors of VCT uptake.

**Results:**

Of 400 study participants, 50.5% were male and 49.5% were female. 93.5% of the respondents were aware of the VCT services, 79.1% had high knowledge on VCT services and 75.9% had positive attitude towards VCT services. On VCT uptake, only 29.3% had ever tested. VCT uptake was found to be significantly predicted by age (p = 0.003), sex (p < 0.001), religion (p < 0.001), exposure to VCT information from a VCT centre (p < 0.001) and type of school ownership (p < 0.013).

**Conclusion:**

Despite high knowledge on VCT services, the uptake of VCT among secondary school students was found to be low. The uptake of VCT was mainly found to be influenced by fear of HIV test results, knowledge and attitude on VCT services, age, education, engagement in sexual relationships, stigma and distance to the VCT centre. Integration of youth friendly VCT services in secondary schools would increase VCT uptake among secondary school students. Support and care received after knowing the test results should be clearly communicated as it helps motivate more young people towards VCT uptake and reduce stigma among them.

## Background

Over 50% of all HIV cases globally are young people aged 10–24 years. Despite high vulnerability to HIV infection, VCT uptake by young people is significantly lower [1]. According to sub-Saharan Africa survey (2005–2010), only 10% male and 15% female of 15–24 years knew their HIV status, implying that majority of the young people in this age are undiagnosed from the HIV epidemic, thus exposing them to high risk of either acquiring or transmitting the disease [1].

In Tanzania, despite high awareness on VCT facilities, 65.8% male and 46.3% female of 15–24 years are not aware of their HIV status and unfortunately, this is where the HIV epidemic is concentrated [2]. In Arusha, 69.8% male and 30.3% female of 15–24 years had never tested [3]. Only 50% female and 39% male in secondary or higher education in Tanzania know their HIV status, giving an average of 45% VCT uptake among them [2]. The low response to VCT among young people is said to be associated with different factors, ranging from fear of knowing their HIV status to the limiting factors towards the service and social issues influencing the attitudes and behaviors of the service providers [1]. However, approximately 50% increase of HIV related deaths among adolescents between 2005 and 2012 was found to be contributed by inadequate friendly VCT services, poor prioritization of adolescent issues, inadequate treatment and lack of support to the young people [4].

Despite high awareness and knowledge on VCT services, the response to HIV testing by young people has been reported to be low [2]. This study aimed at determining the factors that influence young people to uptake VCT so as to influence early HIV detection to avoid increased AIDS cases and risk behaviors, influence support and care to HIV victims [5,6]. Furthermore, facilitate the designing of appropriate strategies by the government and policy makers geared toward increasing VCT uptake among the young people. Since young people are an important force for development, increasing VCT uptake would benefit not only the young people themselves, but also determine the health of the future generation and sustainable economic development of the nation.

## Methods

A cross sectional study that involved quantitative methods was conducted in Arusha City which is among the 7 councils in Arusha region, located in the northern part of Tanzania. The council has 3 divisions, 19 wards and 51 secondary schools of which 23 are public secondary schools and 28 are private secondary schools with a total of 28,000 students in all secondary schools [7].

Students in public and private secondary schools in the City were eligible to be enrolled in the study. However, participants who didn’t agree to participate in the study were excluded at early stages of recruitment. The sample size was determined using an estimated VCT uptake among secondary school students, according to the Tanzania Demographic and Health Survey 2010, 45% of secondary school students had undergone VCT [2]. By assuming 95% confidence interval and 0.05 marginal error, the sample size of 380 was determined using a one sample proportion formula. A non-response rate of 5% was added to give a sample size of 400.

A multi-stage sampling procedure was applied to identify the secondary schools that were involved in the study and stratification sampling method followed by systematic sampling method was used to identify the study participants.

- **Stage 1:** This was done by selecting at random with replacement the two divisions out of three divisions using lottery method.
- **Stage 2:** From each selected division, two wards were randomly selected with replacement by lottery method to give a total of 4 wards out of 19 wards.
- **Stage 3:** From each selected ward, two secondary schools were randomly selected with replacement by lottery method to give a total of 8 secondary schools that were involved in the study out of 51 secondary schools available in the city of which 6 were mixed schools and two non-mixed (one for girls and the other for boys). Stratification sampling technique was used to identify the study participants. Each secondary school was stratified according to the number of classes available for which some schools involved form I-IV, and others form I-VI and for mixed schools, stratification was further done according to sex so that both females and males could fairly contribute to the study. Students from each class were stratified into females and males. Then study participants were selected by systematic method of sampling from each stratum and sub-stratum, whereby participants from each stratum were arranged in ascending order according to the alphabetical order of their names. The sampling interval was calculated for each stratum and/or sub-stratum by dividing the population size of the stratum/sub-stratum with the required sample size of each stratum/sub-stratum. The starting participant was selected at random by lottery. Then participants were selected at the calculated interval until the required sub-sample was obtained in a particular stratum/sub-strata. Similarly, for the single education secondary schools, participants were systematically identified from each stratum (class) until a total sample size of 400 was obtained.

The questionnaire was administered to the study participants by the principal investigator to collect the relevant information. Data collected was coded into the computer, cleaned and then analysed using statistical package for social sciences (SPSS) version 16. Measures of central tendency and dispersion were used to summarize the continuous data and proportions for the categorical data. Analytical statistics using bivariate statistics were done using crude odds ratio (COR) at 95% confidence interval (CI) to measure the strength of association between VCT uptake and independent variables. Any association with p-value < 0.05 was considered significant. Binary logistic regression was used to identify independent predictors of VCT uptake using adjusted odds ratio (AOR) at 95% confidence interval.

Ethical clearance was obtained from the Kilimanjaro Christian Medical University College Research Ethical Committee with a certificate No. 669. Permission to proceed with the study was sought from the Ministry of Education through the Director of Arusha City Council and heads of specific secondary schools where the study was conducted. Consent was sought from secondary school students who were aged 18 years and above. For those students aged below 18 years, assent was sought from the students themselves and consent from the heads of particular secondary schools on behalf of parents but also letters were sent to the parents/guardians of particular students who were aged below 18 years to voluntarily give permission and allow their sons or daughters to participate in the study. Information was kept confidential and identification numbers were used to ensure anonymity.

## Results

Among 400 students, 50.5% were male. The age of the respondents ranged between 13–24 years with a mean age of 16.4 (±1.9) years. Participants were from Form I up to VI. Form I-II constituted 27.8%, Form III-IV, 61% and Form V-VI, 11.2%. Christians comprised 82.8%, Muslims 16.2% and other denominations 1%. 68% were in day schools and 32% in boarding schools. Meanwhile 46.5% were in Public schools, 28.5% in Private schools and 25% in Faith-based schools (Table 1).

**Table 1:** **The distribution of the socio-demographic characteristics of students (n =400)**

**Variable**	**Attribute**	**No. (%)**
Sex:		
	Male	202 (50.5)
	Female	198 (49.5)
Age (years):		
	Mean (±SD)	16.4 ± 1.9
	13 – 15	127 (31.8)
	16 – 18	221 (55.2)
	19 – 24	52 (13.0)
Current form of study:		
	I – II	111 (27.8)
	III – IV	244 (61.0)
	V – VI	45 (11.2)
Religion:		
	Christian	331 (82.8)
	Muslim	65 (16.2)
	Other	4 (1.0)
Type of school		
	Day	272 (68.0)
	Boarding	128 (32.0)
Ownership of school:		
	Government	186 (46.5)
	Private	114 (28.5)
	Faith-based	100 (25.0)

### Proportion of VCT uptake

Of the 400 study participants, only 117 (29.3%) had tested for HIV and received their results.

### Knowledge on VCT services

**Figure 1:**
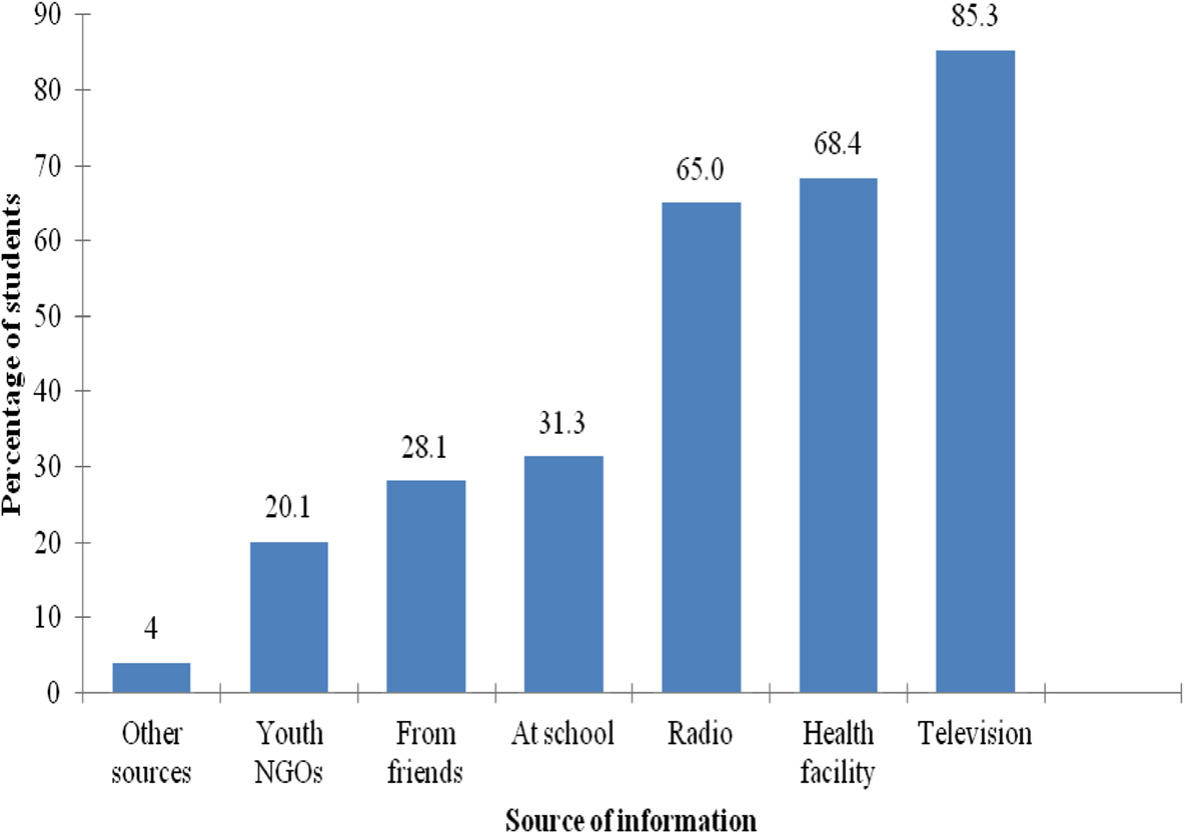
Sources of information for VCT (n = 374).

Among 400 participants, 374 (93.5%) had heard about VCT services, mainly through television, health facilities and radio (Figure 1). Out of these, 309 (82.6%) knew the centres offering VCT services of which 109 (35.3%) reported to have visited VCT centres at least once in the last one year. Of these, 61.3% reported to have visited a health facility, 27.4% stand-alone VCT centre, 7.5% youth organization VCT centre and 3.8% a mobile clinic. Of 109 respondents who reported to have visited VCT centres in the last one year, 64.3% visited the VCT centre purposely for HIV testing, 13.4% for counseling, 20.5% school admission requirement and 1.8% for other purposes (eg. Suspected STDs).

Regarding knowledge on VCT services, among 374 respondents, 296 (79.1%) had adequate or high knowledge on VCT services. This was quantified based on objective judgement, whereby, respondents were classified as adequately knowledged if they could correctly define VCT, mention any VCT centre they know, mention at least three sources of information for VCT services and mention at least 3 important benefits of VCT services for which according to the Ministry of Health and Social Welfare of Tanzania [8] these includes:

To motivate people to change their behaviors to prevent acquisition and transmission of HIV, to reduce anxiety over possible HIV test results, facilitating safe disclosure of test results and planning for future life, improving access to HIV prevention, treatment, care and support services, providing education on reducing stigma and discrimination to HIV victims and prevention of mother to child transmission**.** On the other hand, if one could not give the correct response to any of the above mentioned questions was considered having inadequate knowledge on VCT services [5,8].

#### Sexual activity

When participants were asked to indicate whether they had a sexual partner or not in the last one year, among 374 respondents, 89 (23.7%) reported to be having at least one sexual partner of which 68% were male and 32% were female. On whether they had ever practiced sexual intercourse or not, 73 (19.6%) out of 374 respondents said they had ever practiced. Participants who had at least one sexual partner were further asked to indicate whether they had ever discussed with their sexual partners about getting tested for HIV, 40 (44.9%) out of 89 respondents reported to have discussed.

### Attitude on VCT services

Among 374 respondents, 365 (97.6%) strongly agreed that VCT services are important for prevention of HIV transmission and 370 (98.9%) agreed that it is important to undergo VCT. Out of 370 respondents, 294 (81.4%) said it is important to undergo HIV testing so as to know their health status, protect from HIV infection and plan for future life. Among 257 participants who had never tested for HIV, 195 (75.9%) were willing to undergo HIV testing.

### Association of the socio-demographic characteristics with VCT uptake

Females were about twice significantly more likely to test for HIV than males (OR = 1.8; 95% CI = 1.2-2.8; p = 0.006). Participants aged at least 18 years were significantly 3 times more likely to undergo HIV testing than those who were aged less than 18 years (OR = 2.9; 95% CI = 1.8-4.7; p < 0.001). Likewise, participants who had sexual partners and had discussed with their partners on HIV testing were significantly 3.2 times more likely to undergo HIV test than those who had never discussed (OR = 3.2; 95% CI = 1.3-8.1; p = 0.013). However, participants who had ever practiced sexual intercourse did not significantly differ in VCT uptake from those who had never practiced sexual intercourse (OR = 1.4; 95% CI = 0.9-2.4; p = 0.168).

Participants who were in advanced secondary level were significantly 3 times more likely to test for HIV than those who were in ordinary secondary level (OR = 3.0; 95% CI = 1.6-5.6; p < 0.001). Participants who were taking their studies in private schools were about twice significantly more likely to undergo HIV testing than those who were in public schools (OR = 1.8; 95% CI = 1.1-2.7; p = 0.011). On the other hand, the type of school (boarding or day) did not significantly influence the uptake of VCT among the students p > 0.05 (Table 2).

**Table 2:** **Socio-demographic characteristics versus VCT uptake (n = 400)**

**Variable**	**Total**	**HIV test status**		**OR (95% CI)**	**p-value for the tested**
		**Tested**	**Not tested**		
		**No.(%)**	**No.(%)**		
Sex:					
Male	202	49 (24.3)	153 (75.7)		
Female	198	73 (36.9)	125 (63.1)	1.8 (1.2-2.8)	0.006
Age (years):					
Younger than 18	299	73 (24.4)	226 (75.6)		
18 or older	101	49 (48.5)	52 (51.5)	2.9 (1.8-4.7)	<0.001
Current form of study:					
O’level (Form I-IV)	355	98 (27.6)	257 (72.4)		
A’level (Form V-VI)	45	24 (53.3)	21 (46.7)	3.0 (1.6-5.6)	<0.001
Religion:					
Christian	331	93 (28.1)	238 (71.9)		
Muslim + Other (non- Christian)	69	27 (41.5)	40 (58.0)	1.9 (1.1-3.2)	0.022
Type of school					
Day (single + mixed sex)	272	80 (29.4)	192 (70.6)		
Boarding (single + mixed sex)	128	42 (32.8)	86 (67.2)	1.2 (0.7-1.8)	0.491
Ownership of school:					
Government	186	45 (24.2)	141 (75.8)		
Private	214	77 (36.0)	137 (64.0)	1.8 (1.1-2.7)	0.011
Sexual practice (n = 374)					
No	301	88 (29.2)	213 (70.8)		
Yes	73	27 (37.2)	46 (62.8)	1.4 (0.9-2.4)	0.168
Discussed with a sexual partner about HIV testing (n = 89)					
No	49	10 (20.4)	39 (79.6)		
Yes	40	18 (45.0)	22 (55.0)	3.2 (1.3-8.1)	0.013
Visited VCT centre					
(n = 398)					
No	289	63 (21.8)	226 (78.2)		
Yes	109	59 (54.1)	50 (45.9)	4.2 (2.6-6.8)	<0.001

#### Predictors of VCT uptake

Significant predictors of increased likelihood of VCT uptake were being a female student (p < 0.001), studying in a private school (p = 0.013), aged at least 18 years (p = 0.003), belonging to a non-Christian religion (p < 0.001), exposure to VCT information from a VCT centre (p < 0.001) (Table 3).

**Table 3:** **Factors that significantly predict VCT uptake**

	**Adjusted odds ratio**	**95% C.I**		**p-value**
		**Lower**	**Upper**	
Age: **<18 years**	0.367	0.189	0.714	0.003
Sex of student: **Male**	0.292	0.172	0.495	<0.001
School ownership: **Government**	0.517	0.306	0.871	0.013
Religion of student: **Christian**	0.321	0.171	0.603	<0.001
Visited VCT centre: **Visted**	5.648	3.284	9.714	<0.001

### Factors hindering VCT uptake

**Figure 2:**
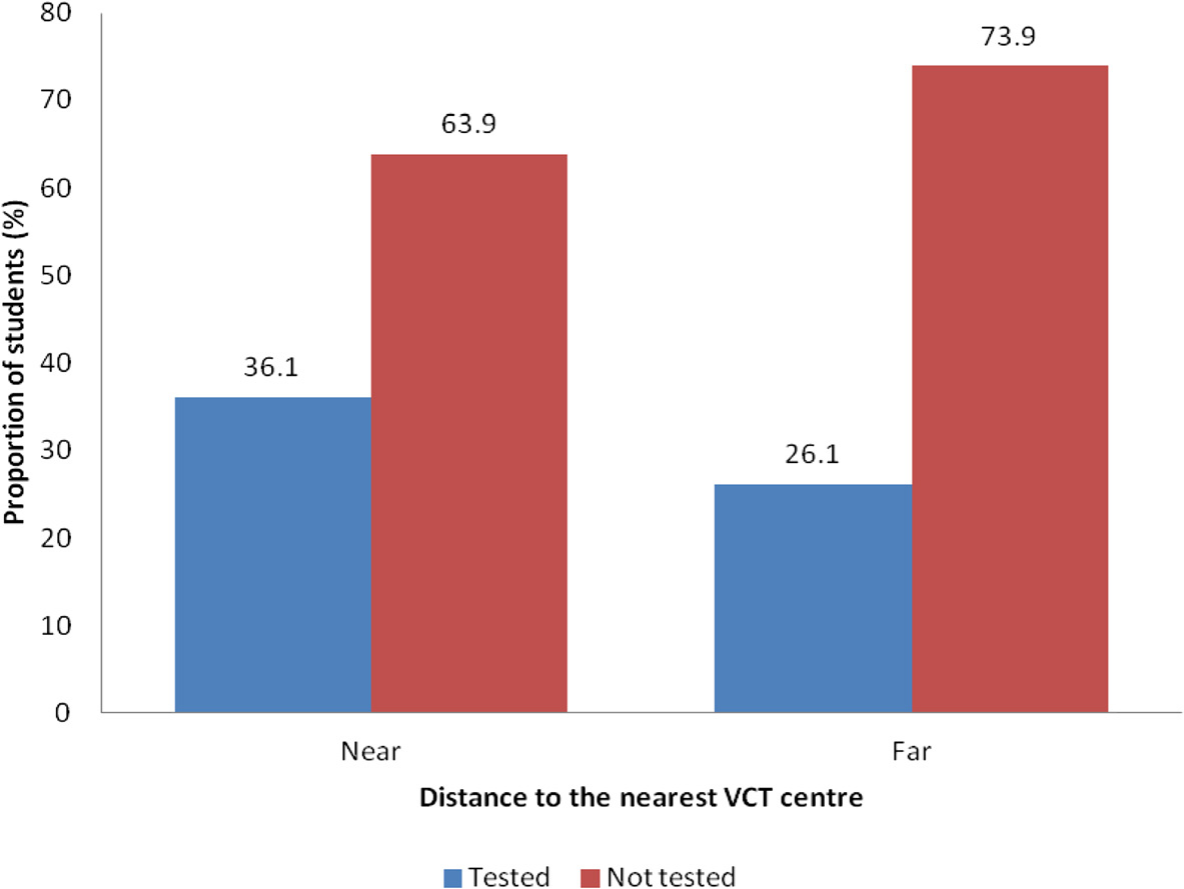
VCT uptake versus distance to the VCT facility (n = 358). p = 0.034; OR =1.6; 95% CI = (1.0-2.5).

**Figure 3:**
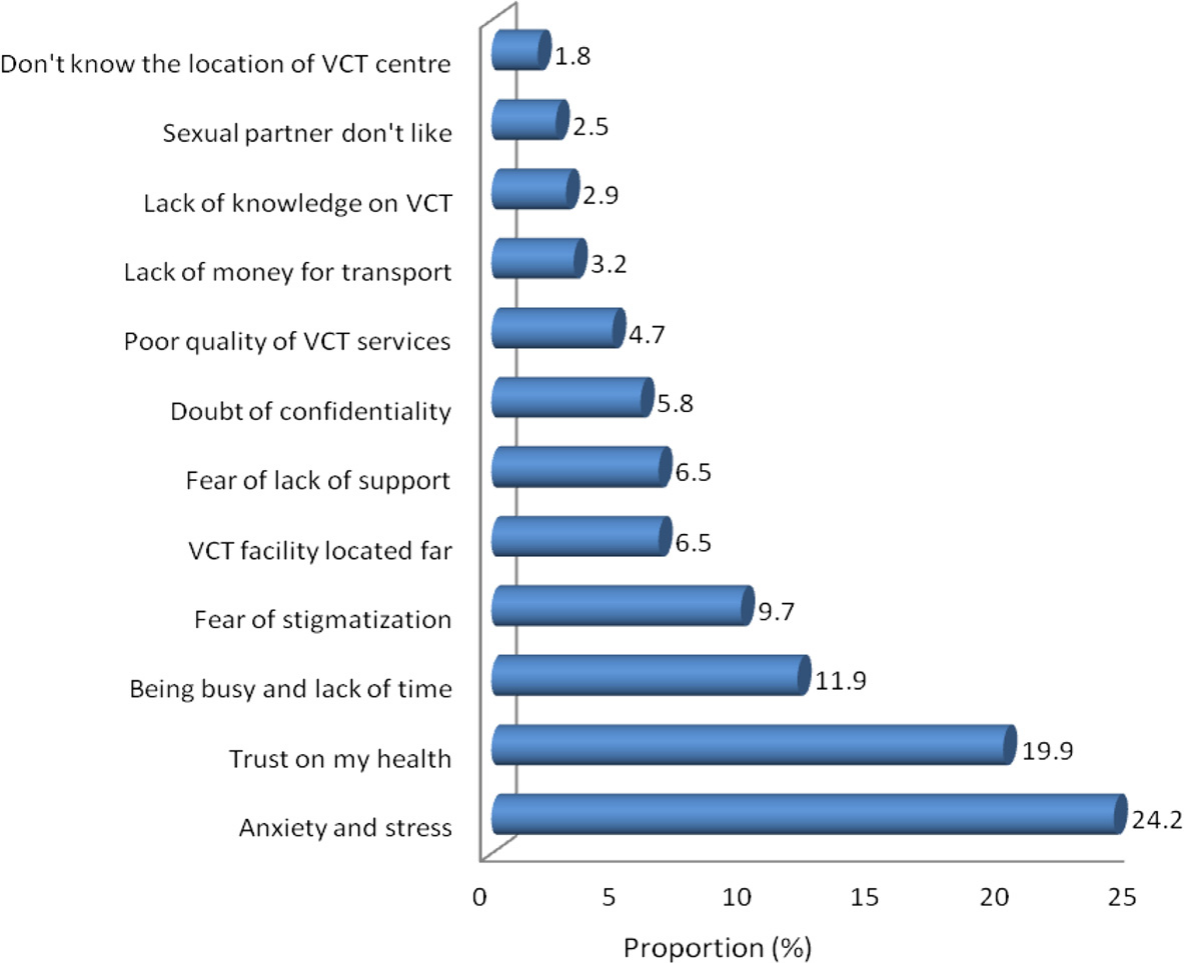
Factors hindering VCT uptake, n = 227; 1 missing).

Among 374 respondents, 358 (95.7%) rated distance as among the factors influencing VCT uptake. Those who reported that, the VCT centre was near their area were 60% more likely to test for HIV than those who rated it as far (OR = 1.6; 95% CI = 1.0-2.5; p = 0.034) (Figure 2). Other factors that were mainly reported to influence the young people towards VCT uptake are as presented in Figure 3.

## Discussion

It is important to measure the coverage of VCT uptake among young people, not only because of their vulnerability to HIV, but also because they may experience some obstacles in accessing the VCT services [2]. According to the findings of this study, females were more likely to uptake Voluntary HIV Counseling and testing as compared to males. This might be due to the fact that females of 15–24 years old tend to start having sexual activity earlier as compared to males [2]. On the other hand, the low uptake of VCT by males may be due to the fact that males are not fully involving themselves in HIV prevention programs, making it hard for them to recognise the importance of knowing their HIV status as compared to females. This finding is similar to a study done in Ethiopia whereby VCT uptake was found to be higher among females as compared to males [9]. But also look similar to a finding in Western Uganda where by males were found to have low VCT uptake due to inadequate involvement in HIV prevention programs [10].

The rate of VCT uptake was found to be higher among those participants aged 18 years and above as compared to those who were aged below 18 years. This finding might be due to the fact that as the young people grow, they are exposed to VCT and HIV education which make them recognise the importance of knowing their health status. This finding is similar to the finding of a study conducted in Cameron whereby VCT uptake was found to increase with age [11]. However, this study differs with the finding of a Tanzania study by Sukari et al., [12] whose findings indicated that VCT uptake decreased with age. The authors argue that as age increases, young people become more sexually active thus hesitating to undergo HIV testing due to fear of the test results. However, this depends also on the age in which the sample started as respondents at extreme ages may have lower response to VCT uptake due to different reasons such as being sexually inactive, low VCT education and others.

The rate of VCT uptake was also found to increase with the level of education. This is because as the level of education increases, students are being exposed to more education on VCT services and HIV infection which provide them with more confidence of undergoing HIV test but also with some skills on how to avoid HIV infection. This finding is similar to a study conducted in Tanzania-Mwanza, whereby VCT uptake was found to increase with increase in the level of education [13]. This finding is also similar to a study done in Cameroon in which VCT uptake was found to increase with the level of education [11]. Other researchers got similar findings to a survey done in Tanzania whereby VCT uptake was found to increase with the level of education [3].

According to this study, the major proportion of the respondents were aware of the VCT services as majority of the respondents had heard about VCT services and the main source of VCT information was reported to be Media (TV and Radio). The findings look similar to a study conducted in Ethiopia in which the major proportion of the respondents were found to be aware of the VCT services with majority of the respondents (75.66%) mentioning TV and radio as their main source of VCT information [9]. This finding might be due to the fact that many of the participants either reside in town or near town areas where sources of information like media and internet services are easily accessible. In addition, health facilities are available and easily accessible in town, which provides the young people with more access to the VCT information through posters and leaflets in the facilities as well as direct health information from the health care providers. The finding of this study shows that, participants who had visited a health facility were more likely to test for HIV than those who had not visited. This might be due to the fact that people often go to a health facility when they already have a health problem, providing them with more chances of getting to know their health status through the health care providers.

The study found that less number of participants received VCT information from their schools compared to media and health facilities. This might be due to the fact that the VCT education programs are not fully given priority in schools enough to be included in the school programs or curriculums and also the frequency of being mentioned by the teachers during class sessions determines the level of VCT awareness among the students. A few respondents reported receiving VCT information from the parents, friends and worshiping places like church and others, implying that, in these key areas VCT services are not yet given priority though in real sense important areas such as worshiping places if given priority may help increase the VCT services awareness and hence improve HIV testing uptake among the young people. These findings also look similar to a study conducted in northern Tanzania among pregnant women whereby partners involvement, parents, and religion were among the factors found to be influencing VCT uptake [14].

The major proportion of the respondents had high knowledge about VCT services. These findings are similar to a study done in Ethiopia whereby the majority of the respondents were found to have adequate knowledge on VCT services [9]. This might also be due to exposure to VCT education among the students either in school or from other areas.

A major proportion of the respondents had positive attitude towards the VCT services. Similarly another study conducted in Ethiopia found that the majority of the respondents had positive attitude towards VCT services [15]. This was realised as majority of the respondents thought that undergoing VCT was of importance to all people and both HIV-positive and HIV-negative individuals get benefits from the HIV test results [16,17]. This is the correct thinking as those who receive the negative results will know how to protect themselves against HIV infections through abstinence, having one safe and faithful sexual partner and practicing safe sex, but also those who receive positive HIV results will know how to behave so as to prevent further transmission and start medication [18,19].

A significant number of the respondents believed that in order to make more progress in the fight against HIV transmission, VCT services are very important to be given priority in terms of the number of facilities offering the VCT service, quality service to be offered by knowledged care providers, good support to the HIV infected people, ensuring confidentiality and stigma reduction by providing education to the community members. Similar finding in a study conducted in Ethiopia by [9]. The fact that more than three quarter of the study respondents were willing to test for HIV indicates that the majority of the participants understand the importance of VCT services and therefore conducive environments and strategic plans for VCT services can attract more youth towards the service.

A significant number of respondents reported fear from anxiety and stress that may result after have came to know that they are HIV positive as among the main reasons towards low VCT uptake. Some respondents even reported that, if the results become HIV positive they would lose focus on their studies and all other future plans. However, this is due to the fact that they lack knowledge and confidence on how they are going to cope with either of the test results, but also they are not sure of the support and care that may be available for them after have tested and received the positive HIV test results. The findings are similar to a study conducted in Ethiopia, whereby fear from anxiety and stress was found to be among the main factors influenced VCT uptake among the young people [9].

Among the prominent reasons for poor response to VCT was fear of stigmatization from the family and community members. A significant number of the study participants said that, they don’t like to go for VCT as they fear from being stigmatized particularly when they are well labeled as HIV victims. This finding is similar to a study conducted in Tanzania-Mwanza, whereby poor response to VCT was associated with fear of stigma [13,20]. This finding also look similar to the one for a study done in Western Uganda, whereby a significant number of respondents reported fear from being stigmatized as being associated with low response to VCT [10]. Because of this young people may lack confidence to cooperate with different people for some key responsibilities and hence getting deprived of some important rights in the society.

A significant number of the study participants considered themselves at low risk of contacting HIV infection. This was realized as some of the respondents thought that it is not possible for them to contact HIV, while others reported to be trusting on their health and therefore they couldn’t find the importance of going for HIV test. Similar finding was observed in a study conducted in Guizhou province-China, whereby low response to VCT uptake was contributed by low risk perception of acquiring the disease [21,22]. Nevertheless, there are different ways one can acquire HIV and even protection with condoms sometimes may not offer hundred percent protection.

Distance to the VCT centre has been found to be among the factors that hindered the decision towards VCT uptake. Participants who reported to reside near the VCT centre were more likely to undergo HIV testing than those who reported to reside far. Among the reasons given included absence of VCT centre around their school premises, lack of knowledge about VCT and its location, lack of money to go for VCT as it involved some costs to reach the centre. But also a significant number of the respondents reported to be busy with no enough time to go for VCT services. Similarly, a study conducted in Tanzania-Mwanza, long distance was associated with low VCT uptake among the students [5,12]. Also a systematic review of published qualitative research in sub-Saharan Africa found that, direct and indirect costs were found to be associated with low uptake of VCT [23,24].

## Study limitation

The study was based on secondary school students. Thus, the findings may not reflect some ideas for those who have never been at school.

## Conclusion

Despite high knowledge on VCT services, the uptake of VCT among secondary school students was found to be low. The uptake of VCT was mainly found to be influenced by fear of HIV test results, knowledge and attitude on VCT services, age, education, engagement in sexual relationships, stigmatization and distance to the VCT centre. Integration of VCT centres or outreach programs in secondary schools and increasing the number of youth friendly VCT centres would increase the uptake of VCT services among the young people. There should be effective involvement of religion leaders and teachers in their working places in encouraging uptake of VCT and provision of counseling services with emphasis on support and care received after knowing the test results to be clearly communicated as it help motivate more young people towards VCT uptake and reduce stigmatization among them.

## References

[1] WHO. HIV and Adolescents guidance for HIV testing and counseling and care for adolescents living with HIV. 2013. Available at: http://apps.who.int/iris/bitstream/10665/94334/1/9789241506168_eng.pdf (accessed on 8/4/2014). 25032477

[2] National Bureau of Statistics (NBS) [Tanzania] and ICF Macro (2011). Tanzania Demographic and Health Survey 2010.

[3] National Bureau of Statistics (NBS), Tanzania Commission for AIDS (TACAIDS), Zanzibar AIDS Commission (ZAC), Office of the Chief Government Statistician (OCGS), and ICF International (2013). Tanzania HIV/AIDS and Malaria Indicator Survey 2011–12: Key findings.

[4] Johns Hopkins School of Public Health. Voluntary Counseling and Testing Rigorous Evidence – Usable Results. 2012. Available at: http://www.jhsph.edu/research/centers-and-institutes/research-to-prevention/publications/VCT.pdf (accessed on 13/3/2014).

[5] Ministry of Health and Social Welfare Tanzania. National Guideline for the Management of HIV and AIDS. 2012. Available at www.nacp.go.tz/site/download/nationalguideline42012.pdf. (Accessed on 10/3/2014).

[6] UNGASS. Progress Reporting;United republic of Tanzania. 2010. Available at: http://www.unaids.org/sites/default/files/en/dataanalysis/knowyourresponse/countryprogressreports/2010countries/tanzania_2010_country_progress_report_en.pdf (accessed on 2/4/2014).

[7] Arusha City Council. Secondary Education, 2014. Also Available at: http://www.arushacc.go.tz/index.php/dummy-link-1/item-with-menu-icon (Accessed on 10/4/2014).

[8] Ministry of Health and Social Welfare Tanzania. Standard Operating Procedures for HIV Testing and Counselling ( htc ) services. 2009. Available at: http://digitallibrary.ihi.or.tz/1933/1/Standard_Operating_procedure.pdf. (accessed on 10/3/2014).

[9] Gatta AA (2011). Knowledge and attitudes towards Voluntary HIV Counseling and Testing services amongst adolescent high school students in Addis Ababa Ethiopia. AOSIS Open Journal.

[10] Bwambale MF, Ssali NS, Byaruhanga S, Kalyango NJ, Karamagi ASC (2008). Voluntary HIV counselling and testing among men in rural western Uganda: Implications for HIV prevention. BMC Public Health..

[11] Mbopi-kéou FX, Haddison EC, Nguefack-Tsagué G, Noubom M, Mbatcham W, Ndumbe PM (2012). Voluntary counselling and testing for HIV among high school in the Tiko health district Cameroon. Pan African Medical Journal.

[12] Sukari O (2007). Barriers and attitudes towards HIV Voluntary Counseling and Testing among Secondary School Pupils of Sengerema in Mwanza. Dar Es Salaam Medical Students’ Journal.

[13] Wringe A, Isidingo R, Urassa M, Todd J, Mbata D, Maiseli G (2007). Trends in uptake of voluntary counseling and testing for HIV in rural Tanzania under widely provision of HIV treatments. Trop Med Int Health.

[14] De Paoli MM, Manongi R, Klepp KI (2004). Factors influencing acceptability of voluntary counselling and HIV-testing among pregnant women in Northern Tanzania. AIDS Care.

[15] Tewabe T, Destaw B, Admassu M, Abera B (2012). Assessment of factors associated with voluntary counselling and testing uptake among students in Bahir Dar University Ethiopia. Ethiop. J. Health Dev..

[16] Baisley K, Doyle AM, Changalucha J, Maganja K, Watson-Jones D, Hayes R (2012). Uptake of voluntary counselling and testing among young people participating in an HIV prevention trial: comparison of opt-out and opt-in strategies. PLoS One.

[17] Pikard JL (2009). HIV Voluntary counselling and testing among kenyan male youth aged 13–15 years:The theory of planned behaviour applied. Masters Abstracts International.

[18] Unicef-Tanzania. Adolescence in Tanzania. 2011. Available at: http://www.unicef.org/infobycountry/files/TANZANIA_ADOLESCENT_REPORT_Final.pdf (accessed on 16/4/2014).

[19] WHO. Global health sector strategy on hiv/aids 2011–2015. 2011. Available at: http://whqlibdoc.who.int/publications/2011/9789241501651_eng.pdf?ua=1 (accessed on 26/3/2014).

[20] Kakoko DCV (2006). Voluntary HIV counselling and testing service uptake among primary school teachers in Mwanza-Tanzania. AIDS Care.

[21] Killewo JZ, Kwesigabo G, Comoro C, Lugalla J, Mhalu FS, Biberfeld G (1998). Acceptability of voluntary HIV testing with counselling in a rural village in Kagera Tanzania. AIDS Care.

[22] Ma W, Detels R, Feng Y, Wu Z, Shen L, Chen F (2007). Acceptance of and barriers to voluntary HIV counselling and testing among adults in Guizhou province-China. AIDS.

[23] Musheke M, Ntalasha H, Gari S, Mckenzie O, Bond V, Merten S (2013). A systematic review of qualitative findings on factors enabling and deterring uptake of HIV testing in Sub-Saharan Africa. BMC Public Health.

[24] Medical Research Council. A review of research among black african communities affected by HIV in the UK and Europe. 2013. Available at: http://www.sphsu.mrc.ac.uk/library/occasional/OP015.pdf (accessed on 22/4/2014).

